# A Multidisciplinary Approach for a Patient With Synchronous Lip and Thyroid Cancer Involving Radiotherapy

**DOI:** 10.1002/cnr2.70564

**Published:** 2026-04-29

**Authors:** Aleksandra Nasiek, Paweł Polanowski, Marek Hamm, Anna Kozub, Barbara Lipka, Agnieszka Kotecka‐Blicharz, Krzysztof Składowski

**Affiliations:** ^1^ 1st Radiation and Clinical Oncology Department Maria Sklodowska‐Curie National Research Institute of Oncology Gliwice Poland; ^2^ Department of Nuclear Medicine and Endocrine Oncology Maria Sklodowska‐Curie National Research Institute of Oncology Gliwice Poland; ^3^ 3rd Radiation and Clinical Oncology Department Maria Sklodowska‐Curie National Research Institute of Oncology Gliwice Poland; ^4^ Department of Periodontal Diseases and Oral Mucosa Diseases, Faculty of Medical Sciences in Zabrze Medical University of Silesia Katowice Poland

**Keywords:** head and neck region, papillary thyroid carcinoma, squamous cell carcinoma, synchronous cancers

## Abstract

**Background:**

Synchronous cancers, defined as malignancies diagnosed concurrently or within 2 months of each other, are rare, with an incidence of 1%–6%. Environmental factors such as tobacco smoking play a significant role in their development. Patients with head and neck squamous cell carcinoma (SCC) are at increased risk of second malignancies, complicating treatment strategies and prognosis. This report presents a rare case of synchronous SCC of the lower lip and papillary thyroid carcinoma (PTC).

**Case:**

A 61‐year‐old male with a history of smoking presented with a 2.5 cm exophytic lesion of the lower lip, diagnosed as keratinizing SCC. A suspicious cervical lymph node was revealed, which intraoperatively demonstrated metastatic PTC. Following partial lip resection and neck dissection, total thyroidectomy was performed, confirming a 1.5 mm PTC focus with micrometastases. The patient underwent adjuvant conventional radiotherapy (RTH) for SCC (66 Gy in 33 fractions) and radioiodine therapy (131‐I) for PTC. Post‐therapeutic scintigraphy identified suspected micrometastases in the iliac bone, prompting further 131‐I treatment. Following completion of therapy, the patient achieved a complete response, with no radiologic or clinical evidence of residual disease from both malignancies.

**Conclusion:**

Synchronous SCC of the lip and PTC are exceedingly rare, lacking standardized treatment guidelines. This case highlights the necessity of a multidisciplinary approach, integrating surgery, RTH, and systemic therapy. Careful postoperative histopathological assessment of lymph nodes may reveal unexpected findings that necessitate further diagnostic work‐up and a multidisciplinary approach, as this can significantly influence staging, treatment decisions, and patient prognosis, representing an important key learning point.

## 1 Introduction

Synchronous cancers are defined as malignant tumors detected concurrently or within a two‐month interval following the initial neoplastic diagnosis. Typically, they are rare, with an incidence ranging from 1% to 6%. Environmental factors including tobacco smoking are one of the main risk factors [1]. Patients with squamous cell carcinoma (SCC) of the head and neck represent an increased risk of developing a second malignancy, which significantly affects their diagnostic process, treatment strategies, and overall survival. Synchronous cancers usually are identified during the initial diagnosis of a primary tumor; however, no specific therapeutic guidelines are applicable [2, 3]. This case report presents a 61‐year‐old patient with synchronous SCC of the lower lip and papillary thyroid carcinoma (PTC) who was qualified for individual management with the employment of surgery, radiotherapy (RTH), and radioactive iodine therapy. The rarity of this dual malignancy significantly complicates both diagnostic evaluation and treatment planning, emphasizing the need for a patient‐specific, multidisciplinary strategy. By documenting this unusual case, we aim to expand the limited available evidence and offer potential guidance for managing comparable clinical challenges.

## 2 Case Presentation

A 61‐year‐old male patient with hypertension, hypercholesterolemia, cerebrovascular accident 12 years ago, and an 18 pack‐year smoking history reported a tumor encompassing the right side of the vermillion border of the lower lip, which has been present for 1 year. He was referred to the Maria Sklodowska‐Curie National Research Institute of Oncology in Gliwice, Poland in April 2024. Physical examination showed a 2.5 cm exophytic lesion encompassing the whole lip thickness without enlarged regional lymph nodes. Histopathological examination revealed keratinizing SCC, G1 (well‐differentiated, grade 1) according to WHO Classification of Head and Neck Tumors, 5th Edition. A computed tomography (CT) scan confirmed the presence of the tumor and additionally identified an asymmetrical, spherical lymph node in group Ia on the right side of the neck, measuring 7 × 6 mm. The patient's case was presented at the Head and Neck Unit, where they were deemed for partial resection of the right side of the lower lip and local cheiloplasty. Treatment decisions were made following consultation within a multidisciplinary team, including head and neck surgeons, endocrinologists, radiation oncologists, radiologists, and pathologists. This process was conducted within a single tertiary referral center, enabling coordinated, team‐based management and ensuring a consistent and integrated therapeutic approach.

The excision included a margin of non‐tumor‐involved tissue confirmed by intraoperative histopathological examination. Lymphadenectomy was performed, including the dissection of submental lymph nodes as well as right‐side lymph nodes from Groups I, II, and III. Histopathological examination showed keratinizing SCC, G2 (moderately differentiated grade 2, WHO classification). The pathological stage was determined on pT2N2b (The American Joint Committee on Cancer; AJCC, 8th edition). Additionally, one of the lymph nodes located in group IV on the right side, measuring approximately 1 cm, revealed in intraoperative examination micrometastasis of PTC. Due to this unexpected diagnosis, the surgical team decided to remove the group IV of the lymph nodes of the right side of the neck. The pathological stage was determined as pTxN1b (AJCC, 8th edition). A postoperative ultrasound of the neck visualized a 5 mm hypoechoic tumor located in the upper pole of the left thyroid lobe. Fine‐needle biopsy showed a benign lesion, classified as category II (The Bethesda System). The patient was qualified for a total thyroidectomy with resection of cervical lymph nodes in group VI along with a nodal biopsy of the left side of the neck. During the procedure, the thyroid gland and the central cervical lymphatic system were resected. Lymph nodes of the neck of the left side were collected intraoperatively, with pathological examination confirming the absence of metastatic involvement. In the postoperative histopathological material, a 1.5 mm tumor of PTC was detected in the right thyroid lobe and a micrometastasis in the central cervical lymphatic system (group VI). The PTC final pathological stage, considering the first operation, was determined as pT1aN1b (AJCC, 8th edition).

After 17 days from thyroidectomy, the patient started adjuvant RTH due to SCC of the lower lip. The dose prescription on the clinical target volume (CTV) involved lymph nodes of the groups Ib‐IV on the left side and Ib‐V on the right side of the neck to a total dose of 50 Gy in 25 fractions (CTV1), the oral cavity and lower lip with lymph nodes of the group II on the right side of the neck to a total dose of 60 Gy in 30 fractions (CTV2), and the tumor bed of the lower lip to a total dose of 66 Gy in 33 fractions (CTV3). During RTH, typical radiation‐induced reactions involving the mucosa and skin in grade III were observed (Common Terminology Criteria for Adverse Events; CTCAE v5.0). Laboratory tests revealed elevated liver parameters (total bilirubin: 39.7 μmol/L [normal range: up to 20.5]; AST: 86 U/L [normal range: up to 34]). Following a hepatology consultation, these abnormalities were attributed to caloric deficiency and significant weight loss during RTH. He was offered parenteral nutrition. Due to intermediate risk factors for recurrence according to the American Thyroid Association (ATA 2015) and the European Society for Medical Oncology (ESMO 2019), the patient required postoperative treatment with radioiodine therapy (131‐I) [4, 5].

**FIGURE 1:**
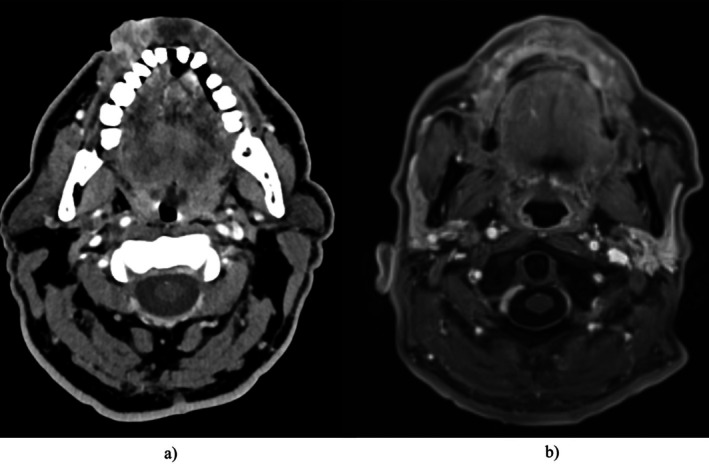
Evaluation using pre‐treatment CT (a) and post‐treatment MRI (b).

Following 43 days after the completion of RTH, the patient was admitted to the Isotope Therapy Unit for adjuvant 131‐I therapy targeting papillary thyroid cancer under exogenous recombinant human TSH (rhTSH) stimulation. During 131‐I treatment, the patient's thyroglobulin level was 0.2 ng/mL under TSH suppression and increased to 0.64 ng/mL on the sixth day of rhTSH stimulation. Post‐therapeutic scintigraphy, supplemented with SPECT–CT (Single‐Photon Emission Computed Tomography), revealed radiotracer uptake in the bed of the pyramidal lobe, the right thyroid lobe, lateral and posterior to the right thyroid lobe (of uncertain character, potentially corresponding to a small lymph node), and in the right iliac plate (without any visible morphological findings in low‐dose CT). Based on these findings, the patient was scheduled for a contrast‐enhanced CT scan of the head and neck, chest, abdomen, and pelvis to evaluate disease advancement. The CT scan showed no radiologic evidence of residual neoplastic tissue. Additionally, no bone lesions suggestive of metastatic involvement were detected, which suggests the presence of a micrometastasis in the iliac bone. The thyroglobulin level during TSH suppression and after stimulation (with a normal anti‐TG concentration) was slightly elevated, consistent with the disease's advancement. Due to the scintigraphic confirmation of metastasis, further treatment with 131‐I was planned. After 6‐months follow‐up, a significant decrease in thyroglobulin concentration was observed (meeting the biochemical criteria for an excellent response according to ATA), and only minimal radiotracer uptake was noted on the post‐therapeutic whole‐body scan in the area of the right iliac bone (with no other pathological uptake). Thyroid cancer treatment was deemed completed and no evidence of recurrence of SCC was found in physical examination and magnetic resonance imaging (MRI) scan. Evaluation using pre‐treatment CT and post‐treatment MRI are presented in Figure 1. At the 12‐month follow‐up (from the completion of 131‐I therapy), there was no evidence of persistent disease based on abdominal ultrasound, chest X‐ray, head and neck MRI findings. However, the thyroglobulin level remained borderline (0.23 ng/mL; target < 0.2). The patient remains under regular oncological monitoring.

## 3 Discussion

Male patients with a history of smoking or alcohol use, as well as those diagnosed with early disease stage and low‐grade primary malignancies, face an elevated risk of developing synchronous or metachronous cancers. The management of such cases presents significant challenges and frequently involves complex therapeutic proceedings. The clinical implications are rarely discussed in the literature. The main challenge is to find a proper therapy strategy that effectively targets both cancers while minimizing toxicity, avoiding significant pharmacological interactions and preserving overall treatment efficacy. Currently, the available evidence is limited to case reports, which must be applied to individual clinical scenarios with caution [6]. Although SCC of the head and neck is one of the most common cancer types in this region and tends to metastasize to the cervical lymph nodes at even early stages, the simultaneous detection of metastatic PTC is a rare occurrence. The estimated incidence of these cancers concurrently is less than 0.5% [7]. A study by Clark et al. confirms that among 1516 patients undergoing treatment for unrelated head and neck cancers, thyroid cancer was accidentally diagnosed in 16 cases, with only two patients presenting lower lip cancer [8]. As a male and a smoker, our patient was at increased risk for SCC of the lip, but the detection of metastases in the cervical lymph nodes of papillary thyroid cancer was highly uncommon.

Surgical resection ensuring safe margins remains the primary treatment for SCC of the lip. If the lymph nodes of the neck are involved, dissection of the appropriate groups should be performed. The use of surgery is supported by many studies, including a retrospective analysis by Japanese researchers who scanned 109 people with SCC of the lower lip. All of them underwent resection by various techniques and patients with clinically detectable lymph nodes underwent neck dissection. The median follow‐up time was 38 months. Recurrence occurred in only 5 responders [9]. A similar view is projected by a 10‐year analysis of 223 patients from Belgrade. Various surgical techniques were used with a median follow‐up time of 56 months. Local metastasis occurred in about 10% of patients, and a mortality rate of 2.2% was recorded [10]. However, surgical treatment can often be insufficient, especially in advanced disease stages or metastases to lymph nodes. Adjuvant RTH plays an important role in such cases. Lang et al. achieved excellent local control and overall survival of 5.2 years with acceptable toxicity. Nineteen patients with SCC of the upper or lower lip treated with postoperative RTH were analyzed. The mean age was 67 years and 58% were male. Fourteen patients underwent neck dissection. All received a median cumulative dose of 66 Gy (range 60–70 Gy) with a median of 2 Gy per fraction (range 1.8–2.2 Gy). No grade III or IV toxicities were observed. Dermatitis and oral mucositis were the most frequently reported adverse effects in Grade 1 or 2 [11]. Our patient initially underwent surgical resection of the tumor with lymphadenectomy. Moreover, adjuvant RTH was implemented to a total dose of 66 Gy in 33 fractions. Treatment was complicated by radiation reaction in grade III, but intensive supportive therapy achieved a satisfactory therapeutic outcome.

PTC is the most common histological type of thyroid cancer. Our patient was first diagnosed with nodal metastasis during intraoperative examination. Preoperatively, the primary tumor focus in the thyroid was not detected. According to National Comprehensive Cancer Network (NCCN) guidelines, the first line treatment in such patients is total thyroidectomy with removal of the affected lymph node compartments and prophylactic central neck dissection, minimum ipsilateral; regional lymph node metastases are most commonly located in the central neck [4, 12]. Approximately 4–12 weeks following thyroidectomy, RAI therapy can be administered but administration within 3 to 12 months post‐surgery is also considered an adequate treatment [13].

This treatment serves a dual purpose: identifying metastatic lesions or residual thyroid tissue, as well as their targeted ablation [14]. An example demonstrating the efficacy of this approach is a comprehensive multicenter study conducted on a cohort of US patients. The analysis revealed that post‐operative RAI therapy significantly reduced both the 15‐year recurrence rate (from 38% to 16%) and mortality rate (from 8% to 3%) when compared to patients managed with observation alone. However, later reports suggest that RAI has the best benefit in patients with regional or distant metastases or cases of incomplete resection with the inclusion of complementary treatment [15, 16]. Our patient received RAI to remnant thyroid ablation, which may help in surveillance for recurrent disease and as adjuvant therapy to try to eliminate suspected and possible micrometastases [12]. Although RAI therapy is typically administered within 6 weeks after thyroidectomy, in this case, it was delayed due to the aggressive course of SCC of the lip, which required priority treatment. Post‐therapeutic scintigraphy, in addition to the typical uptake in the thyroid bed, demonstrated a metastatic lesion in the iliac bone and an indeterminate focus behind the right thyroid lobe bed. Due to the absence of morphological findings on CT and MRI scans, these findings were considered consistent with micrometastases. The comprehensively planned treatment process for two synchronous cancers proved effective because the scintigraphy and MRI scan did not reveal any foci suspected of recurrence of SCC and PTC.

The synchronous occurrence of SCC and PTC in the head and neck region is a rare phenomenon both in clinical practice and in the literature, where it is limited to case reports. Turkish authors described a patient with synchronous SCC of the lower lip and a PTC lesion in the thyroid gland. Unlike our case, both lesions were detected during imaging diagnostics rather than intraoperatively. The patient underwent surgical treatment consisting of tumor excision of the lip and total thyroidectomy. However, no adjuvant therapy was administered, and follow‐up was reported up to 6 months post‐treatment. The patient's subsequent clinical course remains unknown [7]. Another report presented two patients with SCC located in the larynx—differing from our case, where the lesion was on the lip. Both patients underwent radical surgical treatment involving total laryngectomy and thyroidectomy. Additionally, adjuvant RTH was applied in one case. Both patients achieved long‐term disease‐free survival, with follow‐up periods of 2 and 5 years, respectively [17]. None of the described cases included adjuvant 131‐I therapy, which was incorporated in our patient's management according to standard PTC treatment protocols. Available literature describes clinical cases involving either surgery alone [7] or a dual approach combining surgery with adjuvant RTH [17]. None of the published reports have included 131‐I therapy as well. The presented report is distinguished by the sequence of treatment approaches as well as the implementation of all available adjuvant modalities in accordance with current standards. When planning the therapeutic strategy, particular focus was placed on the differences in prognosis between the two malignant tumors. PTC is associated with excellent long‐term survival (approximately 96% at 5 years, 93% at 10 years and over 90% at 20 years) and usually has a milder clinical presentation (mortality rates range from 1% to 6.5%; overall recurrence rates are 15%–35%) compared with SCC [18]. Patient prognosis is also closely associated with disease stage, as defined by the TNM classification [19]. For this reason, PTC treatment was purposely deferred and administered as second‐line therapy following radical surgery for SCC. The use of RTH prior to thyroidectomy would potentially complicate the surgical procedure due to tissue fibrosis and scarring [20]. However, this strategy may also have potential limitations, including delayed definitive treatment of PTC and the complexity of coordinating sequential multimodal therapies. Our adopted strategy enabled the optimization of the treatment course, ensuring effective management of both malignancies while maintaining patient safety. This suggests that our case may be among the first to demonstrate such a comprehensive, multidisciplinary, and coordinated therapeutic approach for synchronous tumors in the head and neck region.

## 4 Conclusions

This case highlights the rarity and diagnostic complexity of two unrelated malignancies. The unexpected intraoperative identification of PTC metastases during surgery for SCC emphasizes the importance of comprehensive histopathological assessment and surgical awareness. It also highlights the need for flexible treatment planning, as in the case of our patient, where therapy was initiated for the more aggressive malignancy to prevent disease progression and enable subsequent treatment of the second cancer type. This approach allowed for an effective response to atypical oncological findings. Synchronous SCC of the lip and PTC is sporadic. Their management is not covered by specific guidelines with uncertain prognostic factors. The treatment regimen presented above exemplifies the positive therapeutic effect leading to prolonged survival. Proper multidisciplinary cooperation allows for effective treatment of the most complex cases.

## Author Contributions

**Aleksandra Nasiek:** conceptualization, formal analysis, methodology, writing – original draft. **Paweł Polanowski:** data curation, supervision, writing – review and editing, formal analysis. **Marek Hamm:** data curation, resources, methodology, writing – review and editing. **Anna Kozub:** formal analysis, data curation, writing – review and editing. **Barbara Lipka:** writing – review and editing, formal analysis, data curation. **Agnieszka Kotecka‐Blicharz:** formal analysis, writing – review and editing. **Krzysztof Składowski:** supervision, writing – review and editing.

## Funding

The authors have nothing to report.

## Ethics Statement

Ethical approval is not required for this study in accordance with local or national guidelines. Written informed consent was obtained from the patient for publication of the details of their medical case and any accompanying images.

## Conflicts of Interest

The authors declare no conflicts of interest.

## Data Availability

All data generated or analyzed during this study are included in this article. Further inquiries can be directed to the corresponding author. The datasets used and analyzed during the current study are available from the corresponding author on reasonable request.
